# Mobile rehabilitation support versus usual care in patients after total hip or knee arthroplasty: study protocol for a randomised controlled trial

**DOI:** 10.1186/s13063-022-06269-x

**Published:** 2022-07-08

**Authors:** Qingling Wang, Sharyn Hunter, Regina Lai-Tong Lee, Xiaofeng Wang, Sally Wai-Chi Chan

**Affiliations:** 1 School of Nursing and Health Management, Shanghai University of Medicine and Health Sciences, Shanghai, China; 2 School of Nursing and Midwifery, The University of Newcastle, Callaghan, New South Wales 2308 Australia; 3 Department of Orthopaedics, Zhongshan Hospital of Fudan University, Shanghai, China; 4 Tung Wah College, Hong Kong, China

**Keywords:** Telerehabilitation, Mobile application, Total hip arthroplasty, Total knee arthroplasty, Self-efficacy, Physical function, Pain, Depression, Anxiety, Health-related quality of life

## Abstract

**Background:**

The global increase in total hip or knee arthroplasty has led to concern about the provision of postoperative rehabilitation. Telerehabilitation may be a strategy to meet the patients’ requirements for rehabilitation after arthroplasty. This study aims to investigate the effectiveness of a telerehabilitation programme delivered via the mobile application WeChat in patients after total hip or knee arthroplasty on the following outcomes: self-efficacy, physical function, pain, depression, anxiety and health-related quality of life.

**Methods:**

This is a single-centre, single-blinded, parallel-group, superiority randomised controlled trial conducted in Shanghai, China. Eighty-four eligible participants who undergo primary total hip or knee arthroplasty will be recruited preoperatively in a university teaching hospital and randomly assigned to the experimental or control group with their informed consent. Once discharged, the control group (*n* = 42) will receive the usual care provided by the hospital. The experimental group (*n* = 42) will receive usual care and a 6-week mobile application rehabilitation programme that consists of physical exercises and techniques for enhancing participants’ self-efficacy for rehabilitation. Baseline assessments will be conducted on the day before hospital discharge, and outcome assessments will be conducted 6 and 10 weeks postoperatively. The primary outcomes are changes in self-efficacy and physical function 6 weeks postoperatively, and the secondary outcomes include pain, depression, anxiety and health-related quality of life. The approach of a generalised estimating equation will be used to analyse the effect of the intervention on outcomes at a significance level of 0.05.

**Discussion:**

This study is the first of its kind conducted in China to incorporate self-efficacy and learning theories as a framework to guide the development of a mobile application rehabilitation programme after arthroplasty. This study will contribute to the knowledge about the effectiveness of mobile application-based rehabilitation among patients after total hip or knee arthroplasty. If the findings are positive, they will support the implementation of mobile application-based rehabilitation in practice, which may potentially increase the accessibility of rehabilitation services as well as patient adherence to rehabilitation.

**Trial registration:**

Australian New Zealand Clinical Trials Registry ACTRN12621000867897. Retrospectively registered on July 6, 2021

**Supplementary Information:**

The online version contains supplementary material available at 10.1186/s13063-022-06269-x.

## Background

Total hip arthroplasty (THA) and total knee arthroplasty (TKA) are two common surgical procedures to treat end-stage degenerative osteoarthritis [1, 2] and primarily occur in older populations [3]. With the ageing population and the increased prevalence of osteoarthritis, the number of THA and TKA procedures has risen globally [4]. Over three million THA and TKA procedures were performed in 2017 around the world [5], which is an increase of 40% compared to the number in 2007 [6, 7]. The involvement of patients in postoperative rehabilitation is important to promote recovery, relieve pain, enhance muscle strength, optimise physical function and improve health-related quality of life (HRQoL) [8, 9].

Rehabilitation services for patients after THA or TKA are conventionally provided face to face at inpatient rehabilitation units or outpatient rehabilitation departments [10, 11]. For people who live in remote areas with limited local health care resources or during a pandemic, such as COVID-19, access to face-to-face rehabilitation services may be difficult [12, 13]. The rapidly growing demand for rehabilitation also challenges the sustainability of face-to-face rehabilitation services [14]. With the advancement in technology, telerehabilitation, which refers to the remote delivery of rehabilitation services via information and communication technologies, such as telephones and computers, has emerged to complement or offer an alternative to face-to-face rehabilitation [15, 16]. Among all these telerehabilitation services, mobile applications (apps) are increasingly being used because they are easy to access [17] and allow health care professionals to provide support whenever and wherever patients need it [18, 19].

The effectiveness of mobile app-based rehabilitation among patients after THA or TKA has been evaluated by several studies conducted in the USA, Australia, Portugal and China [18, 20–25]. These studies delivered rehabilitation services, such as exercise demonstrations, reminders, progress monitoring and follow-up, via mobile apps to the patients at home. These studies supported that mobile app-based rehabilitation, when compared to face-to-face rehabilitation, had the potential to achieve similar outcomes in pain relief as well as in the improvement of physical function, range of motion and HRQoL. However, these studies, except two [18, 25], were quasi-experimental or cohort studies. Evidence derived from randomised controlled trials (RCTs) is essential, as RCTs are considered the gold standard to investigate the effectiveness of interventions [26].

Psychological outcomes were seldom measured in previous studies [27]. Many patients after hip or knee arthroplasty experience psychological problems, such as depression and anxiety [28]. These problems negatively impacted their postoperative outcomes, such as experiencing more pain, lower physical function and higher dissatisfaction [29, 30]. Patients’ self-efficacy is also a critical psychological factor that impacts patients’ motivation, adherence and performance in rehabilitation [31], which would further influence their postoperative physical function and psychological well-being [32]. Thus, in addition to the outcomes that were commonly assessed in previous studies (i.e., pain, physical function, range of motion and HRQoL), psychological outcomes regarding depression, anxiety and self-efficacy should be included in the evaluation of the effectiveness of mobile app-based rehabilitation [11, 32, 33]. Furthermore, the application of a theoretical framework was seldom reported in the development of existing mobile app programmes, which may affect the programmes’ quality and utility [34, 35].

In China, approximately 689,000 THA or TKA procedures are performed annually [36]. The accessibility of face-to-face postoperative rehabilitation services is insufficient, mainly due to the lack of rehabilitation facilities and qualified health care professionals [37, 38]. Therefore, the rehabilitation programmes in some Chinese hospitals consist of written and pictorial instructions provided to the patients at hospital discharge that the patients follow at home instead of attending the clinic for face-to-face rehabilitation [23]. However, patient adherence to this type of  rehabilitation 3 months after surgery was only 23.5% (*n* = 213) because patients did not have support from health care professionals [39]. The wide uptake of smartphones in China has led health care professionals to support patient care at home via mobile apps such as WeChat [23, 24]. Current mobile app programmes mainly focus on diabetes, hypertension and hepatitis [40], whereas those targeting arthroplasty are lacking [41]. Thus, rigorously controlled trials with theory-based interventions are warranted to support evidence-based practice in mobile app-based rehabilitation among Chinese patients after THA or TKA.

This study aims to evaluate the effectiveness of a mobile app rehabilitation programme compared with usual care among Chinese patients after THA or TKA regarding the outcomes of self-efficacy, physical function, pain, depression, anxiety and HRQoL. We hypothesise that compared to the control group receiving usual care, the experimental group receiving the mobile app rehabilitation programme in addition to usual care will report a significant improvement in self-efficacy, physical function and HRQoL as well as a significant reduction in pain, depression and anxiety.

## Methods/design

### Study design

**Fig. 1:**
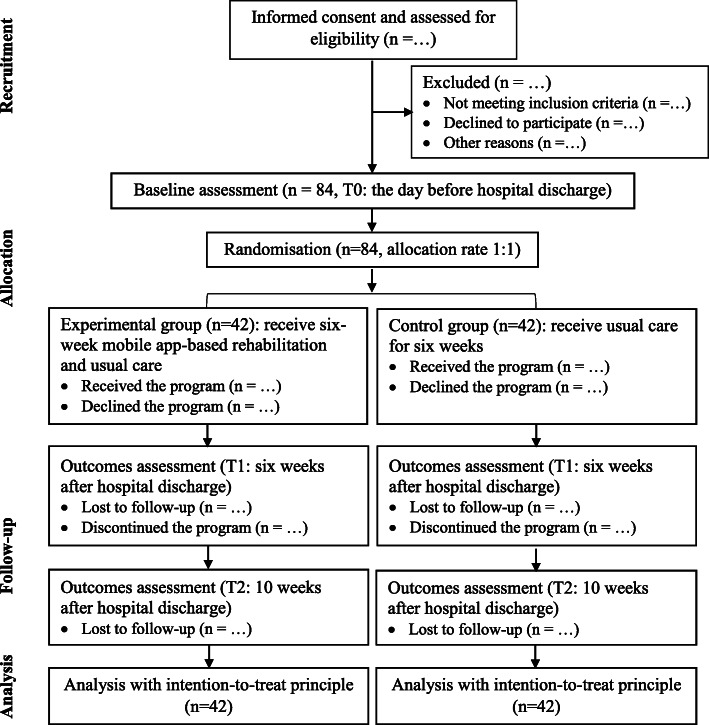
Flow chart of the trial

This is a single-centre, single-blinded, superiority RCT with two parallel groups. Participants undergoing primary THA or TKA will be recruited preoperatively in the orthopaedic wards. They will receive standard inpatient rehabilitation services and the same care during hospitalisation. On the day before hospital discharge, they will complete the baseline assessment (T0) and will be randomly assigned to the experimental or control group. Once discharged, the experimental group will receive a 6-week mobile app rehabilitation programme and usual care, whereas the control group will receive usual care for 6 weeks. Outcomes will be assessed at 6 weeks (T1) and 10 weeks (T2) after hospital discharge. The flowchart of the RCT is presented in Fig. 1. The Standard Protocol Items: Recommendations for Interventional Trials (SPIRIT) checklist is provided in Additional file 1.

#### Sample size

The sample size was calculated using the formula developed by Sugimoto et al. [42], incorporating the co-primary outcomes of this study: physical function and self-efficacy. Based on previous studies in arthroplasty, we expect the effect size of the intervention on self-efficacy to be 1.2 and that on the physical function to be 0.7 [20, 43]. The effect size ratio is approximately 1.7 (1.2/0.7). The correlation coefficient between these two outcomes was estimated to be 0.3 [44]. We set the attrition or nonresponse rate at 20% based on the evidence from the study of Walters et al. [45]. The present study requires a sample size of 84 in all (42 in each group) to achieve a power of 0.8 at a 0.025 level of alpha error.

#### Randomisation

Participants will be randomly assigned to the experimental or control group using simple randomisation with a 1:1 allocation ratio. A person who is not a member of the research team produced the allocation sets using a computerised random number generator. The allocation sequence will be concealed from the researchers using enclosed identical, sequentially numbered, opaque, sealed envelopes, which are impermeable to intense light. After a participant has been enrolled and has completed the baseline assessment, the researcher (QW) will open the next sealed envelope and assign the participant to the indicated group.

#### Blinding

Due to the nature of the intervention, the experimental group will use a mobile app while the control group will not; it is impossible to blind the participants and the researcher (QW) who will deliver the intervention. However, the outcome assessor, a research assistant who has no relationship with the participants, will be blinded to the allocations throughout the study. The researcher (QW) will provide the assessor with assessment training prior to the study. The assessor will independently assess all outcomes for the two groups at T0, T1 and T2. Participants are required in advance not to communicate with the assessor about to which group they are allocated.

### Participants

#### Recruitment

The participants will be recruited from the orthopaedic wards of a 1700-bed, tertiary-level, university teaching hospital in Shanghai, China. Recruitment flyers that contain the researcher’s telephone number will be distributed in the wards. Patients who are interested in the study can contact the researcher (QW) by making a telephone call. The researcher will explain the study and provide the participant information statement. Patients will have 2 days to consider whether to participate in this study. If the patients agree to participate, they will sign the consent form, complete the sociodemographic sheet, complete the baseline assessment and be assigned to either the experimental or control group. We expect that 75% of patients are eligible and 56% of eligible patients will consent to participate in the research [20]. It will take approximately 17 weeks to recruit the required number of participants.

#### Inclusion criteria

The inclusion criteria are (1) adults (greater than or equal to 18 years) after unilateral primary THA or TKA, (2) possess a mobile device (e.g., smartphone or tablet) with an Internet connection, (3) able to access the mobile app WeChat, (4) able to complete the programme and relevant follow-up 3 months after hospital discharge, (5) able to communicate with the researcher in Chinese (Mandarin) and (6) able to provide informed valid consent to participate in the study.

#### Exclusion criteria

Participants will be excluded if they (1) undergo revision and bilateral arthroplasty; (2) have concomitant health conditions that might have interfered with the rehabilitation exercises, such as class II or above heart failure according to the New York Heart Association [46]; (3) received other lower-limb surgery in the last 6 months or will undertake another lower limb surgery within 3 months; (4) have severe vision impairment or blindness according to the International Classification of Disease 11 [47]; and (5) have major postoperative complications, such as incision infection and venous thromboembolism.

### Interventions

Interventions will commence immediately after hospital discharge. The experimental group will receive a mobile app rehabilitation programme on top of usual care, whereas the control group will receive usual care only.

#### Usual care

Usual care of the hospital is based on an expert consensus on enhanced recovery after THA and TKA in China [48]. Home-based rehabilitation exercises are explained to the patients by ward nurses at hospital discharge. Take-home rehabilitation instruction pamphlets are also provided. The home-based rehabilitation exercises consist of 12 exercises for THA and TKA, which aim to improve muscle strength, joint mobility and physical function in daily living. Patients are required to practise rehabilitation exercises according to the instructions for 6 weeks. If they have any questions during rehabilitation, they can telephone the ward nurses. Patients will be followed up when they complete the rehabilitation programme (i.e., 6 weeks after hospital discharge) and 4 weeks later (i.e., 10 weeks after hospital discharge).

#### Mobile app rehabilitation programme

The mobile app rehabilitation is a 6-week programme delivered via the mobile app WeChat, which aims to enhance patients’ self-efficacy for rehabilitation as well as improve their outcomes regarding physical function, pain, depression, anxiety and HRQoL. The design of this programme is guided by two theories, Bandura’s self-efficacy theory [49] and Illeris’ model of learning [50]. The exercise regime used in the mobile app rehabilitation programme is the same as that used in usual care to ensure comparability between the groups. The setting of progressive rehabilitation goals and exercise intensities is based on recent literature [51] and an expert consensus on best practices after hip and knee arthroplasty [11].

Both theories adopted in the present study have been recommended in telehealth research because they may facilitate the creation of effective and interactive interventions [52, 53]. Bandura’s self-efficacy theory guides researchers to adopt strategies that may enhance patients’ confidence in their capability to accomplish rehabilitation tasks. According to the theory, there are four sources of self-efficacy: direct mastery experiences, vicarious experiences, verbal persuasion and arousal state [49]. The present study addresses these four sources with the establishment of achievable small goals as well as the provision of peer sharing, a discussion forum and psychological techniques [31, 54]. Illeris’ model of learning guides researchers to understand the nature of the learning process and the conditions that influence this process [50]. To facilitate the participants’ learning process, the present study uses visual presentations, such as pictures and videos, rather than text messages, and the information is delivered in short segments.

The mobile app rehabilitation programme consists of physical exercises as well as techniques for enhancing participants’ self-efficacy for rehabilitation. The exercises incorporated in the mobile app rehabilitation programme are similar to usual care, and they are demonstrated by videos or photos via WeChat. Participants are encouraged to practise the exercises for 1 h daily and at least 5 days per week [20, 22, 55]. Exercise goals for each week are established, and rehabilitation instructions are arranged in small tasks to help participants achieve the overall exercise goals. Previous patients who have completed the rehabilitation and recovered are invited to share their experiences via the app. An asynchronous discussion forum will be held via WeChat, involving all participants in the experimental group, the researchers, an arthroplasty surgeon, a nurse and a physiotherapist. Participants can post their experiences and questions at any time. The researcher (QW) will respond to the questions within 24 h. Praise, encouragement and reassurance will be provided through discussions and chats. To help relieve stress and anxiety, techniques, such as muscle relaxation exercises, are included in this programme. More details are provided in Additional file 2. The mobile app rehabilitation programme has been reviewed for content validity by a panel that consisted of an arthroplasty surgeon, a nurse, a physiotherapist and two patients.

The mobile app rehabilitation programme will be delivered by the researcher (QW). On the day before hospital discharge, the researcher will teach the participants in the experimental group to use the programme on WeChat. A booklet that illustrates how to use the mobile app rehabilitation programme will also be provided to take home. To improve engagement, the researcher will send a message on Monday of each week to remind the participants to undertake exercises, and the participants will be encouraged to record their use of the programme and completion of the rehabilitation tasks in a paper diary.

#### Concomitant rehabilitation and medications

At the orthopaedic surgeon’s request, some participants may undertake additional in-person rehabilitation sessions during the programme. These participants will be included in the data analysis, but they will be asked to report this concomitant intervention by answering two additional questions on the outcome assessment questionnaires: ‘Have you been required to attend in-person rehabilitation? If yes, how many times?’ Other regular medications the participants take during the programme will also be collected at baseline.

### Outcome measures

**Fig. 2:**
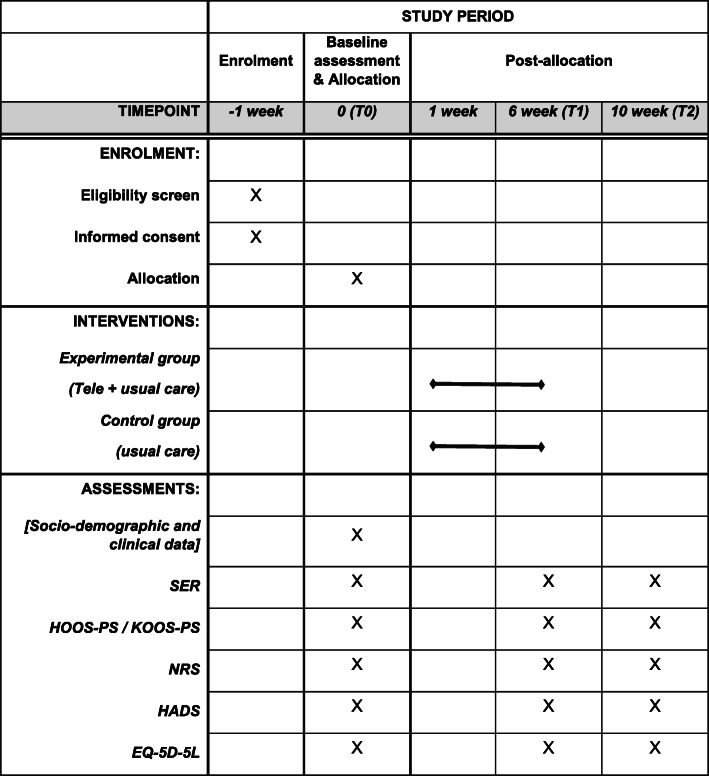
Study schedule of enrolment, interventions and assessments

Data will be collected at three time points: at baseline (week 0, T0), at week 6 immediately after the intervention (T1) and at week 10 as follow-up (T2). Demographic data, including age, gender, living status and education as well as a brief history of health conditions, including surgery, comorbidities and medication, will be collected at T0. The following outcomes will be measured at T0, T1 and T2. Both the experimental and control groups will be assessed by the same assessor using the same measurements and following the same time frame. The assessor will assess the participants via telephone if they are unable to attend the hospital for face-to-face assessment at T1 and T2. The SPIRIT schematic diagram is presented in Fig. 2.

After being recruited, the participants will complete the baseline assessment and then be randomly allocated to either the experimental or the control group. The experimental group will receive a mobile app rehabilitation programme on top of usual care, whereas the control group will receive usual care only. The interventions will last 6 weeks. All outcome measures will be conducted at baseline, completion of the intervention (week 6) and follow-up (week 10). SER Self-Efficacy for Rehabilitation Outcome Scale, HOOS-PS Hip Disability and Osteoarthritis Outcome Score Physical Function Short Form, KOOS-PS Knee Injury and Osteoarthritis Outcome Score Physical Function Short Form, NRS Numeric Rating Scale and HADS Hospital Anxiety and Depression Scale.

#### Primary outcomes

The primary outcomes are changes from baseline in self-efficacy and physical function at the completion of the intervention (T1). The intervention is based on the self-efficacy theory, aiming to improve patients’ physical function after surgery. These two outcomes may provide the most relevant and convincing evidence related to the aim of this study. The study results will be considered positive only if the intervention has an effect on both primary outcomes; thus, no adjustment for multiplicity will be conducted in data analysis [56].

Patients’ self-efficacy for rehabilitation will be measured using the Chinese version of the Self-Efficacy for Rehabilitation Outcome Scale (SER) [57]. The SER is sufficiently reliable and valid to detect patients’ self-efficacy for rehabilitation after hip or knee reconstructive/replacement surgeries [58]. It consists of 12 items that assess individuals’ beliefs in their ability to perform behaviours typical of physical rehabilitation, and each item is rated on an 11-point Likert scale from 0 indicating ‘I cannot do it’ to 10 indicating ‘certain I can do it’ [59]. Higher scores indicate greater self-efficacy.

Patients’ physical function will be measured using the Chinese version of the Hip Disability and Osteoarthritis Outcome Score Physical Function Short Form (HOOS-PS) for patients after THA and the Knee Injury and Osteoarthritis Outcome Score Physical Function Short Form (KOOS-PS) for patients after TKA [60, 61]. The HOOS-PS and KOOS-PS are valid for assessing patients’ perceptions of their hip or knee functions [62]. The HOOS-PS has five items, whereas the KOOS-PS has seven. Each item is graded from 0 indicating ‘no difficulty’ to 4 indicating ‘extreme difficulty’ [60, 61]. The raw sum score can be further converted in two directions: from no difficulty (0) to extreme difficulty (100) or from extreme difficulty (0) to no difficulty (100) [63]. To align with other scales in this study, such as the SER and EQ-5D-5L, the scores will be summed using the nomogram of worst (0) to best (100), with higher scores indicating better physical function. The minimal clinically important difference used in this study is 8 points for the HOOS-PS or KOOS-PS [64].

#### Secondary outcomes

The secondary outcomes are changes from baseline in pain, anxiety, depression and HRQoL at T1 and T2, as well as changes from baseline in self-efficacy and physical function at T2. Adherence-related data will also be collected as supportive secondary outcomes.

Patients’ perceived level of pain will be measured using the Numeric Rating Scale (NRS) [65]. This study will use a horizontal 11-point numerical scale, with 0 representing ‘no pain at all’ and 10 representing ‘pain as bad as it could be’. Participants could select a number (0–10 integers) to reflect the intensity of their pain. The NRS is reliable (*r* = 0.96) with good construct validity (*r* = 0.95 correlated to the visual analogue scale), and it is preferred by the patients with pain over other measures because it is easy to comprehend and complete [65]. To maintain consistency in pain assessment, the NRS will be included in the paper diary, and the participants will mark their pain level daily when they wake.

Patients’ levels of anxiety and depression will be measured using the Chinese version of the Hospital Anxiety and Depression Scale (HADS) [66]. The HADS is a widely used instrument to measure the severity of anxiety and depression among patients in nonpsychiatric clinics [67]. It is a 14-item instrument containing two subscales: anxiety (HADS-A, 7 items) and depression (HADS-D, 7 items). Each item is scored using a 4-point Likert scale ranging from 0 indicating ‘not a problem’ to 3 indicating a ‘high level of problems’ [66]. The subscale scores of the HADS-A and HADS-D can be obtained by summing of individual item scores.

Patients’ perceived HRQoL will be measured using the Chinese version of the EQ-5D-5L [68]. The EQ-5D-5L measures HRQoL on five dimensions: mobility, self-care, usual activities, pain/discomfort and anxiety/depression [69, 70]. In each dimension, respondents can indicate their health by ticking the box next to the most appropriate statement, and a digit number expressing the level selected will be produced [68]. Combining the digits for each dimension will produce a five-digit code that describes the patient’s health state. To compare the health state between the groups, the codes will be converted to index values according to the established value set for China [71]. The participants’ judgement of their health will be recorded with the EQ-VAS, where the endpoints are marked as 100 indicating ‘the best health you can imagine’ and 0 indicating ‘the worst health you can imagine’ [68].

The number of days when the participants use the WeChat programme and complete their rehabilitation tasks will be collected through reviewing rehabilitation diaries. The frequency of participants’ posts in the discussion forum on WeChat will be counted to understand online engagement. Complications and fall incidence that occur after randomisation will be investigated by additional questions on the questionnaire to provide information about the safety of using the app. With the individual’s consent, an interview will be conducted to discuss the benefits and barriers to using the app with participants in the experimental group.

### Data analysis plan

IBM® SPSS Statistics 26.0 will be used to analyse the data [72]. The first author will perform data entry and analysis using participants’ identification codes. Categorical variables, such as gender, will be presented in frequencies and percentages. Continuous variables, such as age, will be presented as means with standard deviations or medians with interquartile ranges. Demographic characteristics and baseline variables will be compared between the two groups. Student’s *t*-tests will be used if the continuous data are normally distributed; otherwise, Wilcoxon rank-sum tests will be used. For categorical data, chi-square tests or Fisher exact tests will be conducted when chi-square testing is not valid. The effectiveness of the intervention will be analysed using the intention-to-treat principle [73]. The generalised estimating equation (GEE) model [74] will be used to compare the differential changes in the outcome variables between the groups across time points at a significance level of 0.05 (two-tailed). The GEE increases the efficiency of the estimates of the parameters in longitudinal trials by accounting for the within-subject correlations in repeated measurements [75]. This model is flexible to use as it relaxes the distribution assumptions of observed data and can accommodate missing data due to dropouts in RCTs [75, 76]. No imputation will be conducted if the proportion of missing data is less than 5% as the GEE can naturally integrate the missing data; otherwise, multiple imputations may be conducted [77].

### Ethical consideration

Ethical approval to conduct the study has been obtained from the Human Research Ethics Committee of the University of Newcastle in Australia (reference number H-2021-0414) and the ethics committee of the study venue (reference number B2021-096R). Patients will be informed that participation is voluntary and that they can withdraw at any time without a reason. The trial will be discontinued for a given participant if the participant withdraws consent, or if the participant develops a condition that has been diagnosed by the clinical practitioner as unsuitable for continuing the rehabilitation programme, such as joint dislocation and severe fall. The conditions would be documented as adverse events and would be reported to the hospital for evaluation and treatment. Identification codes will be used in data collection to ensure participants’ privacy and anonymity. All electronic data will be saved in a password-protected cloud system of the university. The non-digital documentation will be stored in a locked cabinet in the researcher’s office. Data collected in this study will only be accessed by the research team members. Ethics committees at the university and study venue will monitor the implementation of the study and compliance with the ethical codes. Progress reports will be submitted regularly to the ethics committees for the auditing of the completeness, accuracy and timeliness of data collection as well as of the process related to enrolment, consent, eligibility and adherence to the protocol.

### Modification of the protocol

Any modification of the protocol will be discussed and determined by all research team members. Substantive amendments that may impact the conduct of the study or the ethical rigour of the study, such as changes of objectives, eligibility criteria, study procedure and data analysis, will require a formal written modification to the protocol. The protocol will be identified by a unique version number and date. Such amendments would be approved by the ethics committee of the university and the study venue before they are implemented. The modification will be updated on the trial register and notified to participants after it has been approved. Consent to participate in the modified study will be required. Other amendments, such as minor corrections and clarifications that would not affect the conduct of the study, will be documented as a memorandum.

## Discussion

The present study will investigate the effectiveness of a theory-based mobile app rehabilitation programme regarding self-efficacy, physical function, pain, depression, anxiety and HRQoL among patients after THA or TKA. To the best of our knowledge, this is the first trial conducted in China about mobile app-based rehabilitation after arthroplasty that incorporated a self-efficacy and learning theoretical framework. The results will add robust evidence on the effectiveness of the rehabilitation services delivered via mobile apps and will provide insight into the application of these theories in telehealth. The evidence from this study may contribute to clinical recommendations and future research about mobile app-based rehabilitation. If the findings are positive, the evidence from this study will support the provision of mobile app-based rehabilitation to patients after THA or TKA, which might increase the accessibility of rehabilitation services [18] to benefit patients who are not able to attend face-to-face rehabilitation. With the support of mobile apps, patients’ adherence to rehabilitation might be increased as they could obtain support, such as readily accessible information and reminders [78].

The primary outcomes of this study are changes in self-efficacy and physical function because the mobile app rehabilitation programme targets enhancing patients’ self-efficacy for rehabilitation and improving their physical function. The results will provide evidence about patients’ self-efficacy in telerehabilitation after arthroplasty, which has seldom been reported in previous studies. A previous study reported that telephone follow-up significantly improved patients’ general self-efficacy one month after TKA [79], but patients’ self-efficacy in mobile app-based rehabilitation remains unknown. Self-efficacy for rehabilitation plays an important role in the interpretation of the effectiveness of rehabilitation, and it is suggested to be included in the outcome measures [11]. As patients’ recovery is represented by multidimensional factors involving their physical, mental, social and vocational abilities [80], secondary outcomes, including pain, depression, anxiety and HRQoL, will also be assessed in the present study to examine the changes in patients’ symptoms, emotional recovery and quality of life. These outcomes will provide a comprehensive understanding of the effect of mobile app-based rehabilitation after arthroplasty as well as the opportunity to explore the interaction between physical and psychological outcomes.

### Limitations of the study

Given the nature of the intervention, double-blinding is not possible. However, the outcome assessor will be blinded in this study. To reduce the confounding variables caused by different rehabilitation protocols in different hospitals, the present study will be a single-centre trial. This might reduce the generalisation of the findings to a broader population. The outcomes will be assessed using self-reported measures as it is difficult to conduct objective measures after hospital discharge, especially for patients who live in other cities or rural areas outside Shanghai. Due to the time constraints of a doctoral programme, of which this study is part, the follow-up period is limited to 10 weeks. Future studies could include longer follow-up periods, such as 6 or 12 months postoperatively, to assess the long-term effectiveness of mobile app rehabilitation programmes.

### Trial status

Participant recruitment commenced on May 25, 2021 (protocol version 3, dated April 1, 2021). Up to now, 48 participants have been recruited. Recruitment is expected to be completed in October 2021. Findings from this study will be published in peer-reviewed journals and presented at conferences.

## Conclusion

This study will contribute to the knowledge about the effectiveness of mobile app-based rehabilitation among patients after THA or TKA. If the findings are positive, they will support the implementation of mobile app-based rehabilitation in THA or TKA, which might increase the accessibility of rehabilitation services and patient adherence to rehabilitation.

## Supplementary Information

**Additional file 1.** SPIRIT 2013 Checklist: Recommended items to address in a clinical trial protocol and related documents*.

**Additional file 2.** Content of the mobile app-support rehabilitation program.

**Additional file 3.** Model consent form.

## Data Availability

The study protocol will be published in the journal and provided on the Australian New Zealand Clinical Trials Registry. The unidentified datasets generated from this study will be available from the corresponding author upon reasonable request.

## Abbreviations

THA: Total hip arthroplasty
TKA: Total knee arthroplasty
HRQoL: Health-related quality of life
app: Application
RCT: Randomised controlled trials
SPIRIT: Standard Protocol Items: Recommendations for Interventional Trials
HOOS-PS: Hip Disability and Osteoarthritis Outcome Score Physical Function Short Form
KOOS-PS: Knee Injury and Osteoarthritis Outcome Score Physical Function Short Form
SER: Self-Efficacy for Rehabilitation Outcome Scale
NRS: Numeric Rating Scale
HADS: Hospital Anxiety and Depression Scale
GEE: Generalised estimating equation
