# Supplementary material for: Mobile rehabilitation support versus usual care in patients after total hip or knee arthroplasty: study protocol for a randomised controlled trial
Source: Trials. 2022 Jul 8;23:553. doi: 10.1186/s13063-022-06269-x (PMC9264304; doi:10.1186/s13063-022-06269-x)
Supplement: Supplementary file 2 — Additional file 2. Content of the mobile app-support rehabilitation program. [file 13063_2022_6269_MOESM2_ESM.docx]

Additional File 2. Content of the mobile app-support rehabilitation program

| **CONTENTS** | **PRESENTATION** |
| --- | --- |
| **The 1^st^ week, targets:** (1) become familiar with rehabilitating at home with the assistance of the app; (2) consolidate the exercises learnt during hospitalisation by repeating them; (3) learn about precautions after the surgery. | |
| (1) Welcome and orientation   - Introduce the objectives and procedure of the mobile app rehabilitation program. - Recap how to use each component of the program (this has been explained when the participants were recruited). - Remind the participants whom they can contact if they have any questions. | - Text & pictures. |
| (2) Rehabilitation exercises (All for operated leg. Family members or caregivers can provide help if the patient cannot do exercises in the first week)   - Hip abduction/adduction, 3 x 10 reps x 5 days. - Straight leg raise, 3 x 10 reps x 5 days. - Heel slides, 3 x 10 reps x 5 days. - Sitting knee flexion and extension (for TKA), 3 x 10 reps x 5 days. | - Video demonstration with voice explanation. |
| (3) Educational materials: the precautions after THA and TKA. | - Text & pictures. |
| (4) Question and discussion: any question about rehabilitation. | - Discussion forum on app. |
| **The 2^nd^ week, targets:** (1) improve mobility of the operated joint; (2) improve movements in daily activities; (3) learn about muscle relaxation. | |
| (1) Reminder of exercising and keeping the diary: send a message to the participants on the first day of each week, reminding them to keep going on rehabilitative exercises and recording their performance on the diary. | - Individual chats. |
| (2) Rehabilitation exercises   - Standing march and abduction, 3 x 10 reps x 5 days. - Walking using a walker, 3 x 5min x 5 days. - Steps and curbs, 2 x 10 steps x 5 days. - Get in and out of the bed (for THA), 2 reps x 5 days. | - Video demonstration with voice explanation. |
| (3) Educational materials: how to conduct muscle relaxation. | - Text & pictures. |
| (4) Question and discussion. | - Discussion forum on app. |
| **The 3^rd^ week, targets:** (1) improve mobility of operated joint under certain resistance; (2) strengthen muscles of back and lower limbs; (3) improve movements in daily activities. | |
| (1) Reminder of exercising and keeping the diary. | - Individual chats. |
| (2) Rehabilitation exercises   - Walking using a walker or a stick/cane: 2 x 10 min x 5 days. - Steps and curbs: 3 x 10 reps x 5 days. - Bridge: 2 x 10 reps x 5 days. - Clam (for THA, operated leg): 2 x 10 reps x 5 days. - Knee flexion and extension under resistance (for TKA, operated leg): 2 x 10 reps x 5 days. | - Video demonstration with voice explanation. |
| (3) Peer-sharing experiences: a previous patient who has completed the rehabilitation program shares her story about postoperative recovery. | - Text & pictures. |
| (4) Question and discussion. | - Discussion forum on app. |
| **The 4^th^ week, targets**: (1) strengthen muscles of back and lower limbs; (2) gradually resume daily movements; (3) learn about postoperative anxiety and ways to self-regulate. | |
| (1) Reminder of exercising and keeping the diary. | - Individual chats. |
| (2) Rehabilitation exercises   - Calf raises, mini squats and strides: 2 x 10 reps x 5 days. - Walking using a stick/cane: 3 x 10 min x 5 days. - Steps and curbs: 2 x 15 reps x 5 days. - Clam (for THA): 3 x 10 reps x 5 days. - Knee flexion and extension under resistance (for TKA): 3 x 10 reps x 5 days. | - Video demonstration with voice explanation. |
| (3) Educational materials: what is anxiety, why it occurs after surgery, and how to reduce anxiety by breathing in and out slowly. | - Text; - Video demonstration. |
| (4) Question and discussion. | - Discussion forum on app. |
| **The 5^th^ week, targets**: (1) improve movements in daily activities; (2) strengthen muscles of back and lower limbs. | |
| (1) Reminder of exercising and keeping the diary. | - Individual chats. |
| 2) Rehabilitation exercises   - Walking using a single stick/cane or without any aids, 2 x 15 min x 5 days. - Steps and curbs: 3 x 15 reps x 5 days. - Calf raises, mini squats and strides: 3 x 10 reps x 5 days. - Clam (for THA): 3 x 15 reps x 5 days. | - Video demonstration with voice explanation. |
| (3) Peer-sharing experiences: a previous patient who has completed the rehabilitation program record his performance in rehabilitation and share his experiences. | - Video & voice messages |
| (4) Question and discussion. | - Discussion forum on app. |
| **The 6^th^ week, targets**: (1) improve independence in daily activities; (2) improve balance in movements; (3) learn about postoperative depression and available resources of supports. | |
| (1) Reminder of exercising and keeping the diary. | - Individual chats. |
| (2) Rehabilitation exercises   - Walking without aids, 1 x 30 min x 5 days. - Steps and curbs: 4 x 15 reps x 5 days. - Balance/proprioception training: 2 x 10 min x 5 days. | - Video demonstration with voice explanation. |
| (3) Educational materials: what is depression, why it may occur after surgery, and what supports patients can seek. | - Text. |
| (4) Question and discussion. | - Discussion forum on app. |
| (5) Concluding the program.   - Express our thanks to the participant for participating the program; - Remind the follow-up appointments; - Ask if the participant has any questions or would like to make any suggestions. | - Individual chats. |

Note: THA = total hip arthroplasty. TKA = total knee arthroplasty. reps = repetitions. min = minute.
