# Supplementary material for: Mobile rehabilitation support versus usual care in patients after total hip or knee arthroplasty: study protocol for a randomised controlled trial
Source: Trials. 2022 Jul 8;23:553. doi: 10.1186/s13063-022-06269-x (PMC9264304; doi:10.1186/s13063-022-06269-x)
Supplement: Supplementary file 3 — Additional file 3. Model consent form. [file 13063_2022_6269_MOESM3_ESM.doc]

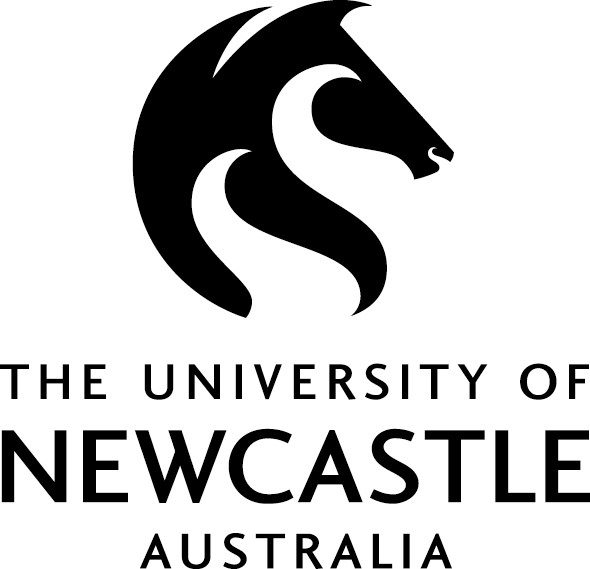
Dr Sharyn Hunter

School of Nursing and Midwifery, University of Newcastle

University Drive, Callaghan, NSW 2308

Tel: 61 2 4921 5957

Sharyn.Hunter@newcastle.edu.au

**Consent Form for the Research Project:**

**App-Based Rehabilitation after Total Hip or Knee Arthroplasty: A randomised controlled Trial**

Qing-Ling Wang, Regina Lai-Tong Lee, Sharyn Hunter, Xiaofeng Wang, Sally Wai-Chi Chan

Document Version [2]; dated [18022021] ID code

I agree to participate in the above research project and give my consent voluntarily.

I understand that the project will be conducted as described in the Information Statement, a copy of which I have retained.

I understand I can withdraw from the project at any time up until the final report is submitted (about 12 months after data collection), and do not have to give any reason for withdrawing.

I consent to:

- being randomly assigned to either the experimental or control group;
- participating in the relevant intervention and the follow-up measures according to the allocation;
- completing the socio-demographic sheet.

I understand that if I have postoperative complications such as incision infection and venous thromboembolism, I cannot continue in the study.

I understand that my personal information will remain confidential to the researchers. Any information that will identify me will be de-identified when making reports and manuscripts.

I have had the opportunity to have questions answered to my satisfaction.

I would like to participate in the telephone interview if I am assigned to the experimental group and the interview will be digitally recorded.

󠄀 YES 󠄀󠄀 NO If tick YES, please provide your phone number so that the student researcher could interview you via the telephone, and please read the following three bullets.

- I understand that the interview will be digitally recorded to make a written copy of my interview information.
- I would like to review the written copy of my interview and give my feedback within two months. 󠄀 󠄀󠄀 YES 󠄀󠄀 NO (if tick YES, please provide your email address below so that the student researcher can send you the copy.)
- I understand that the reporting of this research will quote my comments from the interview. An ID code will be used for the quotes instead of my name.

I would like to receive a copy of the summary of the study results. 󠄀󠄀 YES 󠄀󠄀 NO (if tick YES, please provide your email address below so that the student researcher can send you the copy.)

**Print Name: ___ ______ Telephone number: Email: _____________________**

**Signature: ________________________ Date: ______________________**
